# Comprehensive Physiological and Transcriptomic Profiling of Triploid Pacific Oysters (*Crassostrea gigas*) Under Ammonia Exposure

**DOI:** 10.3390/biology14091121

**Published:** 2025-08-25

**Authors:** Xiumei Liu, Yancheng Zhao, Han Ke, Cuiju Cui, Yanwei Feng, Guohua Sun, Xiaohui Xu, Qiang Wang, Zan Li, Weijun Wang, Jianmin Yang

**Affiliations:** 1 College of Life Sciences, Yantai University, Yantai 264005, China; xiumei0210@163.com; 2 School of Fisheries, Ludong University, Yantai 264025, China; zycheng6633@163.com (Y.Z.); cuicuiju@163.com (C.C.); fywzxm1228@163.com (Y.F.); sgh_smile@163.com (G.S.); xxh83121@163.com (X.X.); ldu_fish@163.com (Q.W.); ladderup@126.com (J.Y.); 3 Shandong Freshwater Fisheries Research Institute, Jinan 250013, China; 7587806@163.com

**Keywords:** triploid *Crassostrea gigas*, ammonia exposure, oxidative stress, immune response, transcriptomics

## Abstract

Ammonia is a common toxic pollutant in aquaculture environments that poses significant threats to the health, growth, and survival of aquatic organisms. This study investigates the physiological and molecular responses of triploid *Crassostrea gigas* to ammonia exposure, focusing on the activation and regulation of oxidative stress and immune-related pathways. By integrating histological observations, biochemical assays, and transcriptomic analysis, we systematically revealed the oxidative stress and immune regulatory mechanisms in the hepatopancreas of triploid *C. gigas* under ammonia exposure. Results showed significant tissue damage in the hepatopancreas, disrupted activities of key antioxidant enzymes including SOD, CAT, and GSH-Px, along with elevated MDA levels, indicating oxidative damage to cellular membrane lipids. Transcriptomic data further indicated significant activation of the glutathione metabolism pathway, with antioxidant genes such as *GPX5* and *GPX7* displaying a dynamic pattern of initial upregulation followed by downregulation, suggesting their critical roles in modulating oxidative stress responses and maintaining cellular homeostasis. Immunologically, ammonia exposure significantly activated lysosomal and phagosomal pathways, as well as multiple signaling cascades including FOXO, mTOR, and PI3K-Akt. Several key immune regulatory genes exhibited dynamic expression changes, reflecting coordinated regulation of apoptosis, autophagy, and energy metabolism to maintain immune defense and cellular homeostasis. Notably, dynamic expression of the *GADD45* gene family in the FOXO signaling pathway underscores the important role of triploid *C. gigas* in mounting stress responses and adaptive immune regulation under ammonia toxicity. This study provides in-depth molecular insights into the integrated response mechanisms of triploid oysters to ammonia exposure, offering a molecular foundation for understanding bivalve adaptation to ammonia and revealing novel perspectives on molluscan ammonia tolerance.

## Simple Summary

Ammonia is a common pollutant in farmed aquatic environments and can seriously harm the health, growth, and survival of animals like oysters. This study looked at how a type of oyster, called the triploid Pacific oyster, responds to ammonia exposure. The researchers found that ammonia caused clear tissue damage in a key digestive organ and disrupted the oysters’ natural defenses, including important protective enzymes. By studying gene activity, they discovered that the oysters first increased certain stress-related genes to fight the damage, but later these responses declined. The oysters also activated genes involved in immune defense and energy use, showing that they were trying to balance cell protection, repair, and survival. One group of genes played a key role in helping the oysters respond to stress and adjust their immune system. This study provides important information on how oysters react to pollution at both the physical and genetic levels. These findings could help improve oyster farming and support the development of pollution-resistant oyster varieties.

## 1. Introduction

Ammonia is a crucial environmental factor in aquaculture systems [1]. In aquatic environments, ammonia exists in two forms in equilibrium: ionic ammonia (NH_4_^+^) and unionized ammonia (NH_3_). The balance between these forms is largely influenced by water pH and temperature [2]. Although both forms are toxic to aquatic organisms, unionized ammonia exhibits markedly higher toxicity due to its greater lipid solubility, which facilitates its penetration across biological membranes [3]. As a result, unionized ammonia can severely impair immune function, disrupt energy allocation, cause respiratory dysfunction, induce oxidative stress, and lead to osmotic imbalance [1,2,3,4,5,6]. It has been reported that millions of tons of ammonia nitrogen-containing wastewater are discharged into aquatic environments annually in China, posing significant threats to the growth and development of aquatic organisms [7]. As a result, the impacts of ammonia on aquatic organisms have attracted considerable research attention. For instance, Cong et al. reported that ammonia exposure significantly increases the early apoptosis rate of gill cells in *Ruditapes philippinarum*, additionally, ammonia was shown to disrupt ATP metabolism and consumption, leading to cellular dysfunction and an exacerbation of apoptotic processes [8]. In a study on chronic ammonia exposure, Ni et al. exposed *Pelodiscus sinensis* to a total ammonia concentration of 6.58 mg/L for 32 days and observed structural damage to gill cells, along with impaired function of key enzymes involved in ammonia transport pathways, including glutamine synthetase (GS) and glutamate dehydrogenase (GDH) [9]. Similarly, research by Chen et al. demonstrated that under high ammonia concentrations (≥12 mg/L), the urea cycle and the metabolism of certain amino acids—such as aspartate and alanine—were significantly suppressed in *Sepia pharaonic* [10]. According to the United States Environmental Protection Agency (USEPA), bivalves are among the most sensitive taxa to ammonia nitrogen, highlighting the importance of assessing its toxicity in this group [11]. Although research on the effects of ammonia nitrogen exposure in bivalves has increased in recent years, studies specifically focusing on *C. gigas* remain scarce. In particular, there is a complete lack of investigations targeting triploid *C. gigas*. Therefore, a systematic evaluation of the impacts of ammonia nitrogen exposure on this species is of great significance for promoting the sustainable development of China’s oyster aquaculture industry.

*C. gigas* is primarily distributed along the Pacific coasts of China, Korea, and Japan. Due to its broad environmental tolerance, rapid growth rate, and high condition index, it has been widely introduced as an aquaculture species across various regions of the world [12]. Triploid *C. gigas*, characterized by sterility, accelerated growth, and the ability to retain high nutritional value during the reproductive season, has become a focus of research and commercial interest [13]. However, mass mortality events of triploid *C. gigas* have been frequently reported during the summer farming season. It is hypothesized that environmental disturbances such as seasonal bottom water hypoxia or sediment resuspension may elevate the concentrations of pollutants, including ammonia nitrogen, thereby contributing to large-scale oyster mortality.

RNA sequencing (RNA-seq) has rapidly advanced due to its high throughput and accuracy, and has been widely applied in recent years to investigate mechanisms such as immune responses and oxidative stress in aquatic organisms [14,15]. In the context of ammonia nitrogen stress, Xiao et al. employed integrated transcriptomic and metabolomic analyses to uncover the stress response and tolerance mechanisms of *Litopenaeus vannamei* under ammonia exposure [16]. Ge et al. used a combined mRNA–miRNA approach to reveal how acute ammonia exposure regulates hepatopancreatic metabolism in *Cyclina sinensis* [17]. Similarly, Zhang et al. utilized transcriptomic analysis to demonstrate that ammonia exposure can disrupt innate immune function in *Corbicula fluminea* [18].

In this study, the molecular mechanisms underlying ammonia nitrogen stress in triploid *C. gigas* were investigated for the first time using transcriptomic analysis. By integrating histological observations and antioxidant enzyme activity assays, we further explored the oxidative stress and immune response mechanisms induced by ammonia exposure from a physiological perspective. The findings of this study will provide theoretical support for the healthy aquaculture and sustainable development of *C. gigas*, and contribute to a more comprehensive assessment of the ecological risks of environmental ammonia to bivalve species.

## 2. Materials and Methods

### 2.1. Collection of Triploid C. gigas and Ammonia Exposure Experiment

Triploid *C. gigas* specimens used in this study were obtained from a commercial aquaculture farm located at Kongtong Island, Yantai, Shandong Province, China. A total of 120 three-year-old oysters were selected, with an average wet weight of 375.65 ± 30 g and shell length of 78.68 ± 7.57 mm. Prior to the experiment, all oysters were acclimated in holding tanks for one week under controlled conditions: temperature at 25 ± 0.5 °C, pH at 8.5 ± 0.1, salinity at 32 ± 0.5, and dissolved oxygen maintained above 5 mg/L. Oysters were fed daily, and the water was renewed daily. To eliminate feeding-related variability, feeding was suspended 48 h before the ammonia exposure trial. For the exposure experiment, three 160 L tanks were prepared, each containing 20 oysters, totaling 60 triploid *C. gigas*. Ammonia nitrogen concentration was adjusted to 10 mg/L by adding ammonium chloride solution. Prior to treatment, nine oysters were sampled as the control group. All other environmental parameters were kept consistent with the acclimation phase. The ammonia concentration in the water was measured and adjusted every 6 h to maintain the target level throughout the exposure period.

### 2.2. Sample Collection

During the experiment, triploid *C. gigas* specimens were randomly sampled from each tank at three time points: 0 h (C_0h), 6 h (N_6h), and 48 h (N_48h). At each time point, three oysters were selected from each tank, totaling nine oysters per time point. Three biological replicates were established, with each replicate consisting of three oysters. Portions of the hepatopancreas tissues were excised and immediately flash-frozen in liquid nitrogen, then transferred to a −80 °C ultra-low temperature freezer for storage. The remaining hepatopancreas tissues were fixed in Bouin’s solution (Ricca Chemical, Arlington, TX, USA) for subsequent histological sectioning and microscopic analysis.

### 2.3. Histopathological Observation

Hepatopancreas tissues of triploid *C. gigas* were fixed in Bouin’s solution for 12 h, followed by trimming and rinsing with purified water. The samples were then dehydrated through a graded ethanol series and embedded in paraffin. Tissue sections of 6 μM thickness were prepared and stained with hematoxylin and eosin (H&E). Finally, the stained sections were sealed with neutral resin for microscopic examination.

### 2.4. Measurement of Physiological Parameters

Hepatopancreas tissues were collected at 0 h, 6 h, and 48 h from each experimental group. According to the kit instructions, the tissues were homogenized on an ice-water bath at a ratio of 1:9 (weight [g]: volume [mL]). The homogenates were centrifuged at speeds specified by each respective assay kit. The resulting supernatants were used to measure the activities of antioxidant-related enzymes, including superoxide dismutase (SOD), catalase (CAT), glutathione peroxidase (GSH-Px), and the level of malondialdehyde (MDA). All assay kits were obtained from Nanjing Jiancheng Bioengineering Institute (Nanjing, China).

### 2.5. RNA Extraction, Library Preparation, and Sequencing

RNA extraction, library construction, and sequencing were performed by Novogene Co., Ltd. (Beijing, China). Briefly, total RNA was isolated from the hepatopancreas tissues of triploid *C. gigas* using TRIzol reagent (Invitrogen, Waltham, MA, USA) following the manufacturer’s protocol. The extracted RNA was divided into two aliquots: one used for library preparation and the other reserved for qPCR validation. RNA quality was rigorously assessed using the Agilent 2100 Bioanalyzer (Agilent Technologies, Santa Clara, CA, USA), RNA with RIN < 7 was re-extracted until it was qualified. Libraries were constructed using the NEBNext^®^ Ultra™ RNA Library Prep Kit for Illumina^®^ (New England Biolabs, Ipswich, MA, USA), and sequencing was conducted on the Illumina NovaSeq platform [19,20,21,22].

### 2.6. Differential Gene Identification, Functional Annotation, and Enrichment Analysis

Raw sequencing reads were initially processed to remove adapter sequences, reads containing more than 10% ambiguous bases, and low-quality reads. The resulting clean reads were aligned to the *C. gigas* reference genome using HISAT2 software (version 2.2.1). The clean reads were mapped to the *C. gigas* reference genome vN1 (GCA_011032805.1), which has been deposited in NCBI. The corresponding annotation file used for gene mapping and functional analysis is not yet publicly available. Differentially expressed genes (DEGs) were identified with DESeq2 (v1.38.3), applying thresholds of *p* ≤ 0.05 and |log_2_ fold change| ≥ 1. These DEGs were then annotated by mapping onto public databases including SwissProt, KEGG, and GO to determine their putative functions and gene identities. Finally, GO and KEGG pathway enrichment analyses were conducted using the DAVID online platform (https://david.ncifcrf.gov/tools.jsp. accessed on 19 October 2024) [23].

### 2.7. Quantitative RT-PCR Analysis

To validate the transcriptome sequencing results, qPCR was performed on a subset of DEGs. Specific primers for qPCR were designed using Primer Premier 5.0 software (Table 1). Due to its stable expression, elongation factor 1-alpha (EF-1α) was selected as the internal reference gene. Relative expression levels of target genes normalized to EF-1α were calculated using the 2^(−ΔΔCT)^ method [24].

## 3. Results

### 3.1. Histological Observation of the Hepatopancreas Following Ammonia Exposure

**Figure 1:**
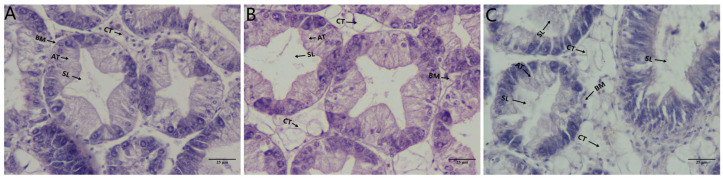
The histopathological sections of the hepatopancreatic tissue of triploid *C. gigas* after ammonia exposure. (**A**): control group at 0 h (C_0h), (**B**): ammonia exposure at 6 h (N_6h), and (**C**): ammonia exposure at 48 h (N_48h). AT: acinar tubules, SL: lumen, BM: basement membrane, CT: connective tissue.

Histopathological sections of the hepatopancreas from triploid *C. gigas* under ammonia exposure are presented in Figure 1. At 0 h (control group), the hepatopancreas exhibited intact tissue architecture, with tightly and orderly arranged tubular glands, clearly defined luminal structures, an intact basement membrane, and normal connective tissue without abnormalities. After 6 h of ammonia exposure, although the overall tissue structure remained relatively intact, signs of tubular disorganization emerged, including widened intertubular spaces and dilated lumens. The basement membrane was largely preserved, but localized vacuolation was observed in the connective tissue. Following 48 h of exposure, severe damage to the hepatopancreas tissue was evident. Cellular arrangement became highly disordered, lumens were abnormally dilated, boundaries between tubules blurred, fragmented tissue debris appeared within the lumens, the basement membrane was partially disrupted, and pronounced vacuolation was present in the connective tissue.

### 3.2. Changes in Hepatopancreatic Physiological Parameters Following Ammonia Exposure

**Figure 2:**
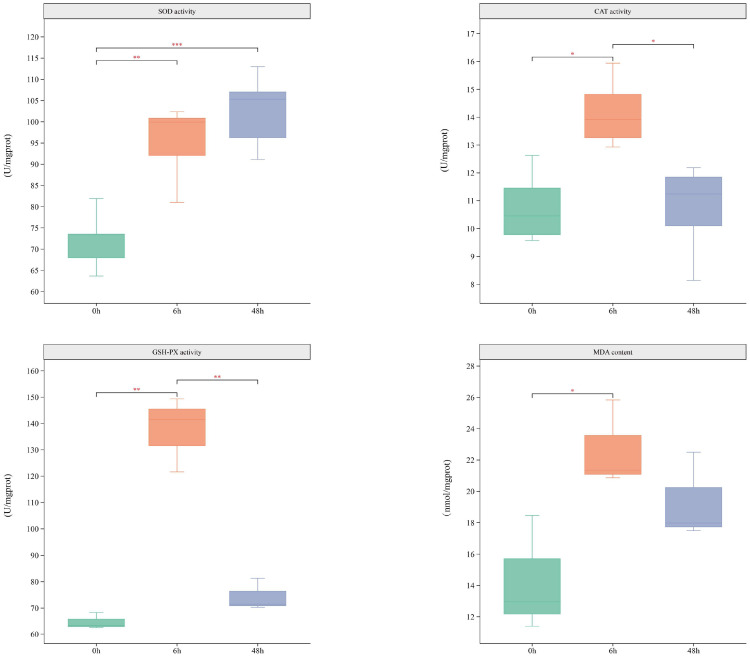
Physiological analysis results. Asterisks indicate levels of statistical significance: *p* < 0.05 (*), *p* < 0.01 (**), and *p* < 0.001 (***).

The activities of antioxidant enzymes SOD, CAT, GSH-Px, and the level of MDA at different time points after ammonia exposure are presented in Figure 2. We found that all four antioxidant indicators including SOD, CAT, GSH-Px, and MDA were significantly elevated at 6 h. Subsequently, the activities of CAT, GSH-Px, and MDA began to decline between 6 and 48 h. Notably, SOD activity continued to increase significantly throughout the 48 h exposure period.

### 3.3. Transcriptome Sequencing Results

RNA-seq was performed on triploid *C. gigas* samples from both the control and treatment groups. Detailed sequencing metrics are presented in Table 2. On average, 62.39% of the clean reads were successfully mapped to the reference genome. The mapped genes were subsequently annotated by aligning them against public databases including NR, NT, SwissProt, GO, KO, and KOG to infer their putative functions.

### 3.4. Differential Gene Expression Analysis

**Figure 3:**
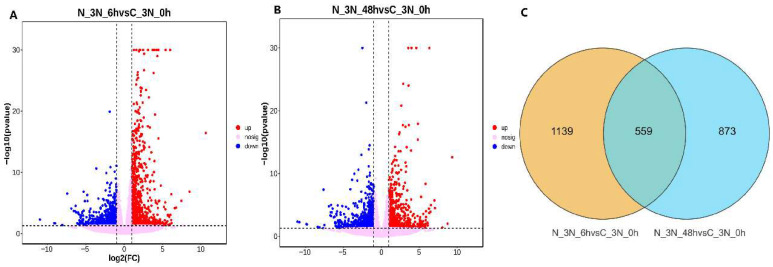
(**A**) Volcano plot of the distribution trend of DEGs between N_3N_6h and N_3N_0h samples. (**B**) Volcano plot of the distribution trend of DEGs between N_3N_48h and N_3N_0h samples. Dots in this graph denote the genes. Red dots correspond to DEGs with upregulated expression; bule dots correspond to DEGs with downregulated expression, and pink dots indicate non-significant genes; (**C**) Distribution of DEGs between two groups.

Compared to the control group (0 h), 1698 DEGs were identified at 6 h post ammonia exposure, including 986 upregulated and 772 downregulated genes. At 48 h, 1432 DEGs were detected, with 677 upregulated and 755 downregulated (Figure 3A,B). Venn diagram analysis revealed a total of 2571 DEGs across both time points, among which 559 DEGs were shared and differentially expressed at both 6 and 48 h (Figure 3C).

### 3.5. Enrichment Analysis

**Figure 4:**
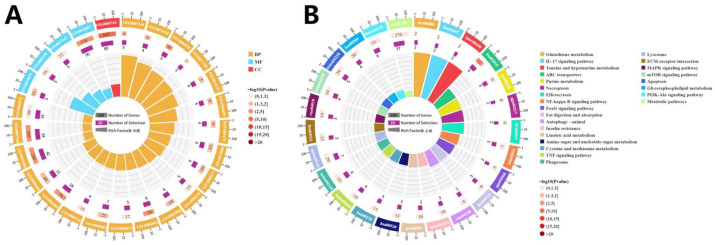
(**A**) GO enrichment circular plot. (**B**) KEGG enrichment circular plot. The four concentric circles in the plot, arranged from outermost to innermost, represent different aspects of the enrichment analysis. The outermost circle displays the enrichment categories, with tick marks indicating gene counts and different colors representing distinct functional classifications. The second circle shows the number of background genes in each category along with their corresponding q-values. The third circle illustrates the number of DEGs in each category. Finally, the innermost circle represents the Rich Factor.

**Figure 5:**
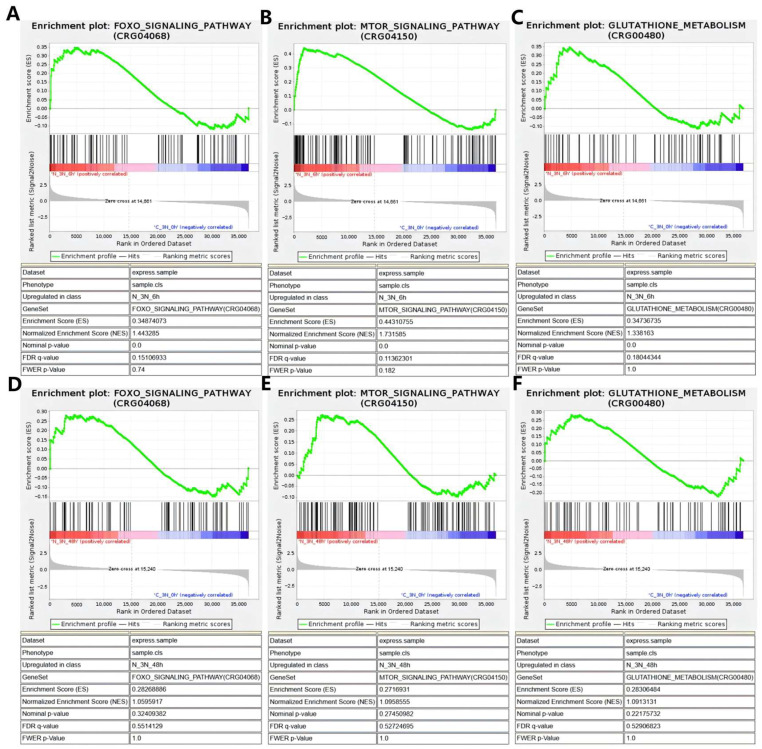
GSEA enrichment analysis plots are shown for key pathways: (**A**) FOXO signaling pathway (6 h vs. 0 h), (**B**) mTOR signaling pathway (6 h vs. 0 h), (**C**) glutathione metabolism pathway (6 h vs. 0 h), (**D**) FOXO signaling pathway (48 h vs. 0 h), (**E**) mTOR signaling pathway (48 h vs. 0 h), and (**F**) glutathione metabolism pathway (48 h vs. 0 h). The green curve at the top represents the enrichment score, indicating the cumulative change in enrichment as genes are ranked; the black vertical lines in the middle denote the positions of the pathway’s genes within the ranked gene list. The gray bar graph at the bottom displays the ranking metric scores for each gene, where the red region indicates genes upregulated in the experimental group, and the blue region represents genes upregulated in the control group.

**Figure 6:**
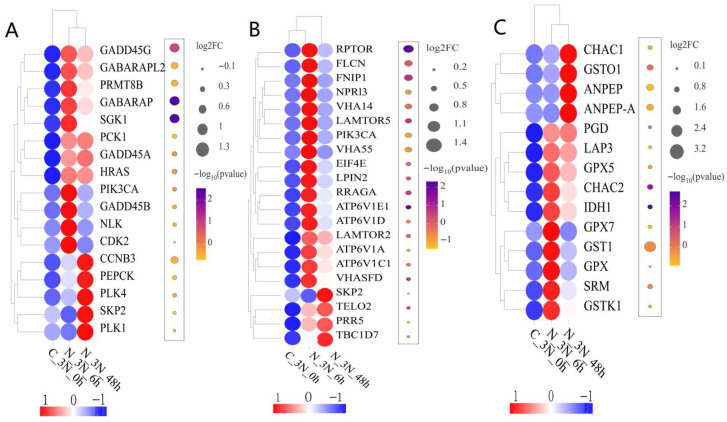
Combined heatmap and bubble plots: (**A**) FOXO signaling pathway, (**B**) mTOR signaling pathway, and (**C**) glutathione metabolism pathway. In the heatmap, color represents normalized expression levels; in the bubble plot, bubble size indicates logFC values, and color represents *p*-values.

GO enrichment analysis (Figure 4A) categorized the DEGs into three main groups: biological processes, cellular components, and molecular functions. The top 30 significantly enriched terms were selected, many of which are closely associated with ammonia exposure. KEGG pathway enrichment analysis (Figure 4B) revealed several pathways related to oxidative stress and immune responses were significantly enriched, indicating that ammonia nitrogen exposure induced notable alterations in antioxidant defense and immune regulation in triploid *C. gigas*. Furthermore, gene set enrichment analysis (GSEA) was performed on pathways with strong relevance and significance to oxidative stress and immunity, including the FOXO signaling pathway, mTOR signaling pathway, and glutathione metabolism pathway, with results shown in Figure 5. The selected genes were visualized using combined heatmaps and bubble plots, as presented in Figure 6, followed by detailed analysis of these genes.

### 3.6. qRT-PCR Validation of DEGs

**Figure 7:**
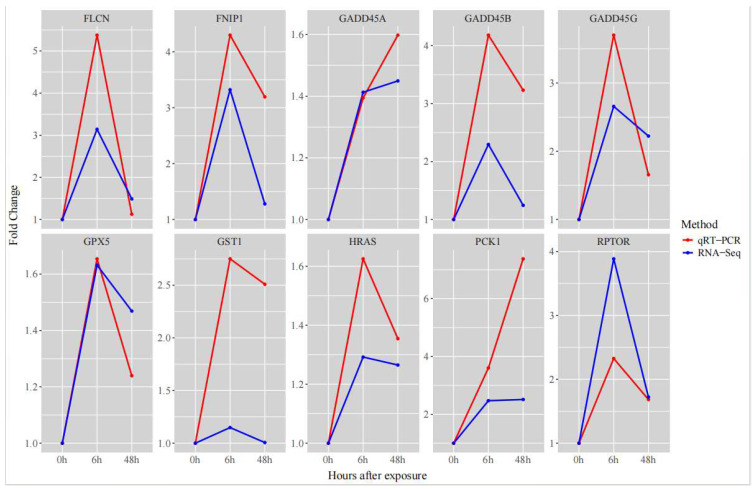
Key DEGs validated by qRT-PCR and RNA-seq. EF-1α was used to normalize gene expression levels in both qRT-PCR and RNA-seq. The *x*-axis represents ammonia exposure time, and the *y*-axis indicates fold change.

We used qRT-PCR to validate genes associated with ammonia exposure. The results (Figure 7) showed similar expression trends compared with the RNA-seq data, confirming the reliability of the RNA-seq analysis [25,26].

## 4. Discussion

Our integrated analysis revealed that ammonia exposure induced significant oxidative stress, disrupted the structural integrity of hepatopancreatic tissue, and impaired both the antioxidant and immune systems, potentially leading to functional damage and immune dysregulation in the hepatopancreas. The combined evidence from histological observations, enzymatic activity assays, and transcriptomic profiling strongly supports the proposed mechanisms of ammonia-induced toxicity, which are elaborated in the following sections.

### 4.1. Tissue Damage Induced by Oxidative Stress

Previous studies have demonstrated that exposure to high concentrations of ammonia disrupts the redox balance in aquatic animals, leading to oxidative stress and compromising the integrity of biological membranes [27], which is consistent with our histological observations of the hepatopancreas in triploid *C. gigas*. Histopathological results indicated that tissue damage became increasingly severe with prolonged ammonia exposure. It has been reported that elevated ammonia levels in water can accumulate in various tissues of aquatic organisms, triggering the release of ROS, which in turn induces oxidative stress and functional impairment [28]. In this study, SOD activity exhibited a sustained and significant increase following ammonia exposure, while the activities of CAT and GSH-PX were significantly elevated from 0 to 6 h and began to decline thereafter, between 6 and 48 h. SOD catalyzes the conversion of O_2_^−^ into H_2_O_2_ and O_2_, and the resulting H_2_O_2_ is subsequently decomposed into harmless H_2_O and O_2_ by CAT and GSH-PX. The increase in SOD activity observed in this study may have contributed to elevated intracellular H_2_O_2_ levels, thereby inducing CAT and GSH-PX activity to maintain redox balance [29]. Nrf2, as a central transcription factor in cellular antioxidant defense, dissociates from its inhibitor Keap1 under oxidative stress, translocates to the nucleus, and binds to antioxidant response elements (AREs), thereby activating the expression of antioxidant enzyme genes, including SOD [30]. Nrf2 was significantly upregulated at 6 h post-exposure, accompanied by an increase in SOD activity following ammonia exposure, suggesting that Nrf2 likely mediates the early upregulation of SOD activity by binding to antioxidant response elements (AREs). After 6 h, Nrf2 expression declined but remained significantly higher than at 0 h, indicating its continued role in regulating SOD expression [31]. In contrast, the activities of CAT and GSH-PX significantly increased during the early phase of oxidative stress (within the first 6 h) but gradually declined thereafter, suggesting that sustained stress may inhibit the activity of CAT and GSH. Besides Nrf2, hypoxia-inducible factor 1-alpha (HIF-1α) also participates in regulating these antioxidant enzymes [32]. Under normoxic conditions, HIF-1α is rapidly degraded; however, during oxidative stress or hypoxia, it stabilizes, translocates into the nucleus, and dimerizes with HIF-1β to activate transcription of antioxidant genes containing hypoxia response elements (HREs) [33]. Studies have shown that HIF-1α can upregulate antioxidant genes, including GPX3 and SOD2, thereby enhancing the cell’s ability to scavenge reactive oxygen species and maintain redox homeostasis [34]. Notably, our transcriptome data show that HIF-1α remains consistently upregulated up to 48 h post-exposure, suggesting its continued regulatory role during the later stages of oxidative stress. However, prolonged oxidative stress may impair cellular energy metabolism and weaken transcriptional regulation, ultimately leading to a decline in antioxidant enzyme expression and activity. Meanwhile, sustained oxidative stress can cause mitochondrial dysfunction, reducing ATP production, which limits the synthesis and maintenance of antioxidant enzymes such as CAT and GSH-PX, thereby suppressing their activities [35]. However, the elevated MDA levels indicate the presence of persistent oxidative damage, suggesting that the antioxidant defense system may not have been sufficient in eliminating the accumulated H_2_O_2_ completely, leading to lipid peroxidation [36]. This hypothesis is further supported by both histological observations and antioxidant enzyme activity assays, which together confirm that ammonia exposure disrupted the antioxidant homeostasis in the hepatopancreas of triploid *C. gigas*, ultimately resulting in oxidative damage.

Transcriptomic analysis revealed that the glutathione metabolism pathway was activated following ammonia exposure. Glutathione, a key component of the cellular antioxidant system, is a major low-molecular-weight free thiol tripeptide that is widely present in animal cells and essential for various physiological processes. It plays a critical role in cellular defense by directly or indirectly scavenging exogenous substances and ROS, thereby protecting cells from oxidative stress-induced damage [37,38]. GSEA enrichment analysis revealed a positive activation of the glutathione metabolism pathway, with key contributing genes including *GPX5*, *GPX7*, and *GPX*. The GPX family constitutes a major group of antioxidant enzymes that reduce ROS levels [39]. *GPX5* facilitates the elimination of hydrogen peroxide and plays a critical role in the physiological response to oxidative stress [40]. *GPX7*, a highly conserved member of the GPX family, functions as an antioxidant enzyme working alongside SOD to convert superoxide radicals into water [41]. Heatmap analysis revealed a dynamic expression pattern of *GPX5*, *GPX7*, and *GPX*, characterized by an initial upregulation followed by a subsequent decline over time. During the early phase of ammonia exposure, the upregulation of these glutathione peroxidase genes suggests that triploid *C. gigas* may enhance cellular defense against oxidative stress by activating these enzymes, thereby mitigating ROS accumulation and the associated cellular damage. As exposure duration prolonged, the expression of these genes gradually decreased, which may reflect a partial alleviation of the initial oxidative insult and a transition to a later regulatory phase of stress response. Moreover, this expression pattern may indicate that under prolonged ammonia exposure, the organism regulates its antioxidant capacity by modulating the expression of GPX family genes. The observed dynamic changes suggest that these genes play crucial roles not only in the initial phase of oxidative stress but also potentially contribute to later-stage stress adaptation and the restoration of cellular homeostasis, warranting further investigation.

This study systematically elucidated the oxidative damage mechanisms induced by ammonia exposure in the hepatopancreas of triploid *C. gigas* through histological examination, physiological parameter assessment, and transcriptomic analysis. The findings demonstrated that ammonia exposure disrupted hepatopancreatic tissue architecture and triggered a pronounced oxidative stress response, characterized by alterations in the activities of key antioxidant enzymes including SOD, CAT, and GSH-PX, accompanied by elevated MDA levels, indicating lipid peroxidation of cellular membranes. Further transcriptomic profiling revealed significant activation of the glutathione metabolism pathway, underscoring its vital role in oxidative stress defense. The expression patterns of *GPX5*, *GPX7*, and *GPX* exhibited a dynamic trajectory with initial upregulation followed by downregulation, suggesting an early protective mechanism against rapid ROS accumulation and a subsequent adjustment phase associated with prolonged stress adaptation or feedback regulation of the antioxidant system. These findings not only highlight the critical involvement of *GPX5*, *GPX7*, and *GPX* genes in the ammonia-induced stress response but also provide essential molecular insights into the environmental adaptability and oxidative damage repair processes of triploid oysters.

### 4.2. Induction of Immune Response

Invertebrate aquatic animals such as mollusks lack adaptive immunity and primarily rely on innate immune mechanisms to defend against environmental stressors [42]. Lysosomes play a pivotal role in innate immunity by regulating inflammatory responses. Additionally, lysosomes participate in the uptake and degradation of macromolecules through endocytosis, phagocytosis, and autophagy [43,44,45]. Phagocytosis serves as a critical innate immune defense and maintains homeostasis by eliminating apoptotic cells and pathogens [46]. Previous studies have reported that ammonia exposure significantly impairs phagocytic activity and lysosomal function in *Sebastes schlegelii* [47]. In the present study, KEGG pathway analysis revealed significant enrichment of lysosome- and phagosome-related pathways, while GO enrichment highlighted terms associated with lysosomal function, phagocytosis, and pathogen recognition. These findings suggest that ammonia exposure activates immune-related pathways in triploid *C. gigas*, potentially reflecting an enhanced cellular endocytic and pathogen clearance response to environmental stress. However, aberrant or sustained activation of such immune responses may disrupt immune homeostasis, supporting the hypothesis that ammonia exposure could detrimentally affect the oyster’s immune defense system.

Transcriptomic analysis also identified several immune-related pathways, including the FOXO signaling pathway, mTOR signaling pathway, PI3K-Akt signaling pathway, NF-κB signaling pathway, and MAPK signaling pathway.

The FOXO signaling pathway, as a critical intracellular signal transduction mechanism, plays a vital role in immune defense [48]. GSEA enrichment analysis of the FOXO pathway revealed its positive activation, with several key contributing genes predominantly upregulated. Among these, the growth arrest and DNA damage-inducible 45 (*GADD45*) family is noteworthy; these proteins interact with DNA demethylases to promote DNA demethylation, thereby regulating diverse cellular processes including oxidative stress response, DNA damage repair, apoptosis, proliferation, differentiation, and inflammation [49]. The *GADD45* family comprises three small, highly acidic proteins: *GADD45A*, *GADD45B*, and *GADD45G* [50]. *GADD45A* is known to induce growth arrest and apoptosis, while *GADD45B* and *GADD45G* similarly play crucial roles in innate immune responses [51,52]. Furthermore, *GADD45A* and its family members modulate various cellular activities—such as growth arrest, differentiation, oxidative stress, cell survival, and apoptosis—through their involvement in NF-κB and MAPK signaling pathways in response to diverse extracellular stimuli [53]. In this study, *GADD45A*, *GADD45B*, and *GADD45G* genes exhibited varying degrees of upregulation within the FOXO signaling pathway, suggesting their crucial roles in modulating cellular immune responses of triploid *C. gigas* under ammonia exposure. Previous research has demonstrated that *GADD45* family members not only participate in DNA repair and cell cycle regulation but also coordinate immune responses, inflammation modulation, and cell survival through interactions with NF-κB and MAPK signaling pathways. The NF-κB pathway serves as a central regulator of inflammation, responding to diverse environmental stressors by inducing the expression of antioxidant and immune-related genes, while the MAPK pathway plays a key role in regulating cell proliferation, differentiation, and apoptosis. *GADD45* proteins act as molecular bridges facilitating signal transduction between FOXO, NF-κB, and MAPK pathways, enabling coordinated intracellular multi-pathway responses [54,55,56,57,58]. Thus, the observed expression changes in *GADD45* genes likely reflect a complex regulatory network activated by triploid *C. gigas* to mitigate cellular damage induced by ammonia stress and to maintain cellular homeostasis and immune defense. Further investigation into the molecular crosstalk among these pathways will provide deeper insights into the adaptive mechanisms and dynamic immune regulation in bivalves facing environmental challenges.

The mTOR signaling pathway regulates a wide range of fundamental biological processes, including cell growth, survival, proliferation, protein synthesis, autophagy, and metabolism. In immunology, mTOR serves as a critical regulator of immune function and is essential for innate immune responses in immune cells. Studies have demonstrated that mTOR plays a central role in sensing and integrating diverse signals from the immune microenvironment, thereby shaping immune cell functions and metabolic states [59,60,61]. In this study, GSEA enrichment analysis identified key contributing genes such as *RPTOR*, *FLCN*, and *FNIP1*. *RPTOR*, a core component of the mTOR pathway, is a highly conserved regulator of cell growth and metabolism that responds actively to nutrient and energy availability. As a pivotal regulator of mTORC1, *RPTOR* plays a significant role in immune responses by modulating immune cell metabolism, functional differentiation, and stress responses, thus maintaining immune homeostasis and coordinating immune effectors [62,63]. The expression changes in these genes under environmental stressors such as ammonia exposure may reflect an integrated mechanism in triploid *C. gigas* that coordinates energy metabolism and immune regulation to cope with physiological challenges posed by adverse conditions. Previous studies have demonstrated that *FLCN* plays a positive role in autophagosome formation, while autophagy contributes actively to immune regulation and cellular homeostasis, constituting an essential component of the innate immune response [64,65]. The *FNIP1* gene plays a crucial role in regulating energy metabolism as well as cell growth and division. Coordinating nutrient and energy availability with cell growth and proliferation is essential for the proper development and function of immune cells [66,67]. Heatmap analysis revealed that *RPTOR*, *FLCN*, and *FNIP1* exhibit a dynamic expression pattern in triploid *C. gigas*, characterized by an initial upregulation followed by downregulation. We speculate that under ammonia exposure, *RPTOR*, *FLCN*, and *FNIP1* may synergistically regulate mTORC1, jointly participating in stress responses related to energy metabolism, autophagy activation, and immune modulation in triploid *C. gigas*. The potential interplay among these genes provides novel insights into the molecular mechanisms by which oysters maintain cellular homeostasis and functional adaptation in high-ammonia environments. Further functional studies are necessary to elucidate the precise regulatory relationships within this network.

The PI3K-Akt signaling pathway plays a central role in immune regulation by controlling immune cell proliferation, differentiation, and migration [68]. Previous studies have demonstrated that the PI3K-Akt pathway in mollusks is involved in modulating immune responses [69,70]. For instance, this pathway can regulate phagocytosis during immune responses triggered by external stimuli, as well as inducing immune defense mechanisms and controlling apoptosis and growth of immune cells following environmental challenges [71,72]. In this study, we observed activation of the PI3K-Akt pathway, with significant enrichment of the *CASP9* gene, a cysteine protease crucial in the mitochondrial apoptosis pathway and an important immune-related gene [73]. Notably, *CASP9* expression was markedly downregulated in triploid *C. gigas* following ammonia exposure, suggesting inhibition of mitochondrial-mediated apoptosis. This finding implies that triploid *C. gigas* may modulate *CASP9*-mediated apoptotic signaling to moderately suppress mitochondrial apoptosis, thereby maintaining tissue homeostasis and mitigating cellular damage induced by ammonia stress.

In summary, ammonia exposure not only disrupts oxidative homeostasis in triploid *C. gigas* but also perturbs multiple key pathways involved in innate immunity. The activation of immune-related signaling pathways, including lysosome, phagosome, FOXO, mTOR, and PI3K-Akt, along with significant changes in the expression of associated immune genes, highlights the broad impact of ammonia stress on immune defense, autophagy, and energy metabolism. Triploid *C. gigas* appears to dynamically regulate essential biological processes such as apoptosis, autophagy, and metabolic adaptation by modulating key genes within these pathways, including *CASP9*, *RPTOR*, *FLCN*, and *FNIP1*. This coordinated, multi-pathway regulatory response may help preserve cellular function and tissue integrity under environmental ammonia stress, offering important molecular insights into the immune adaptation strategies of bivalves under complex environmental challenges.

## 5. Conclusions

This study systematically elucidated the mechanisms of oxidative damage and immune disruption induced by ammonia exposure in triploid *C. gigas* through histological observations, biochemical assays, and transcriptomic analyses. The results demonstrated that ammonia stress caused structural damage to the hepatopancreas, triggered oxidative stress responses, and led to alterations in antioxidant enzyme activities (SOD, CAT, and GSH-PX), along with elevated levels of lipid peroxidation marker MDA, indicating a disruption of the redox balance. Transcriptomic analysis revealed positive activation of the glutathione metabolism pathway and dynamic expression of *GPX5*, *GPX7*, and *GPX*, confirming the oyster’s active response to oxidative stress. Additionally, significant enrichment of immune-related pathways, such as lysosome and phagosome, as well as key signaling pathways including FOXO, mTOR, and PI3K-Akt, was observed. Differential expression of multiple core stress- and immunity-related genes (*GADD45A*, *GADD45B*, *GADD45G*, *CASP9*, *RPTOR*, *FLCN*, and *FNIP1*) further supports the presence of adaptive immune modulation in response to ammonia exposure.

## Figures and Tables

**Table 1:** List of primers used for quantitative RT-PCR validation.

Gene Name	Forward Primer (5′-3′)	Reverse Primer (5′-3′)	Amplicon Length (bp)
* FLCN *	GCTTCCATTGTGAGGTCTGTCTTC	GATTCTTCGTACTGGTGACTGTAAGG	95
* FNIP1 *	CGTCTTGGGTCAGTGGGTCAG	ATAGGTGGAGGGTTTAGAGAGTTCAG	90
* GADD45A *	AAGGTGGACAGCGAGGATAAGG	GGACAGGTCGTCTGGTTCATTG	81
* GADD45B *	GCAGGGAACACAGGATAACACTTG	TGGGTTTCCTCTGGGCACTTAC	93
* GADD45G *	TCGAACGACGTTTGGCATCTATG	GCACTGTTACATCTCCATTGTCTCC	107
* GPX5 *	CTCTCTGCTCCTTCTCCCCTTC	TCCATCCAAATCCACAGTTTCCAAG	118
* GST1 *	ACGATGGTGGTGACGGCTAC	TGGTCTGGTCAGTGATGTCATAAATC	81
* HRAS *	TGACGGAATATAAATTGGTGGTTGTTG	ATGGTTCTGGATAAGTTGGATGGTTAG	80
* PCK1 *	AGGCTGAAGGATGTGGATGGAC	CTGAGATGCGATGCTCTGATATGG	102
* RPTOR *	GTCGCAGACAAGGACGGAATATG	TCTTGGTGTTCTTCAGGTTCTCATTG	91

**Table 2:** Sequencing results.

Samples	Raw Reads	Clean Reads	Q20 (%)	Q30 (%)	GC (%)	Mapping Rate (%)
C_3N_0h_1	47,046,994	45,502,698	99.35	97.96	43.3	61.07
C_3N_0h_2	42,016,988	40,673,606	99.27	97.68	43.05	59.85
C_3N_0h_3	42,767,888	41,345,928	99.34	97.94	43.18	60.57
N_3N_6h_1	42,038,274	41,093,296	99.37	98.01	43.15	60.36
N_3N_6h_2	40,957,084	39,851,994	99.37	98.01	43.49	62.18
N_3N_6h_3	43,731,464	42,477,644	99.4	98.08	43.35	63.29
N_3N_48h_1	42,761,402	40,853,772	99.36	97.96	43.17	66.27
N_3N_48h_2	46,679,872	43,752,672	99.37	98.02	42.71	62.13
N_3N_48h_3	40,195,832	39,153,134	99.39	98.02	43.94	65.75
Average	43,132,866.44	41,633,860.44	99.36	97.96	43.26	62.39

## Data Availability

Data will be made available on request.

## Abbreviations

The following abbreviations are used in this manuscript:

* C. gigas *	* Crassostrea gigas *
GO	Gene Ontology
KEGG	Kyoto Encyclopedia of Genes and Genomes
DEGs	Differentially expressed genes
GSEA	Gene set enrichment analysis
